# Genetic Influences on Behavioral Outcomes After Childhood TBI: A Novel Systems Biology-Informed Approach

**DOI:** 10.3389/fgene.2019.00481

**Published:** 2019-05-22

**Authors:** Brad G. Kurowski, Amery Treble-Barna, Valentina Pilipenko, Shari L. Wade, Keith Owen Yeates, H. Gerry Taylor, Lisa J. Martin, Anil G. Jegga

**Affiliations:** ^1^Division of Physical Medicine and Rehabilitation, Cincinnati Children's Hospital Medical Center and Departments of Pediatrics and Neurology and Rehabilitation Medicine, University of Cincinnati College of Medicine, Cincinnati, OH, United States; ^2^Department of Physical Medicine & Rehabilitation, University of Pittsburgh School of Medicine, Pittsburgh, PA, United States; ^3^Division of Human Genetics, Department of Pediatrics, Cincinnati Children's Hospital Medical Center and University of Cincinnati College of Medicine, Cincinnati, OH, United States; ^4^Division of Physical Medicine and Rehabilitation, Department of Pediatrics, Cincinnati Children's Hospital Medical Center and University of Cincinnati College of Medicine, Cincinnati, OH, United States; ^5^Departments of Psychology, Pediatrics, and Clinical Neurosciences, University of Calgary, Calgary, AB, Canada; ^6^Abigail Wexner Research Institute at Nationwide Children's Hospital, Department of Pediatrics, The Ohio State University, Columbus, OH, United States; ^7^Division of Human Genetics, Department of Pediatrics, Cincinnati Children's Hospital Medical Center and University of Cincinnati College of Medicine, Cincinnati, OH, United States; ^8^Division of Bioinformatics, Cincinnati Children's Hospital Medical Center and Department of Pediatrics, University of Cincinnati College of Medicine, Cincinnati, OH, United States

**Keywords:** traumatic brain injury, genetics, systems biology, pediatrics, behavioral outcomes

## Abstract

**Objectives:** To test whether genetic associations with behavioral outcomes after early childhood traumatic brain injury (TBI) are enriched for biologic pathways underpinning neurocognitive and behavioral networks.

**Design:** Cross-sectional evaluation of the association of genetic factors with early (~ 6 months) and long-term (~ 7 years) post-TBI behavioral outcomes. We combined systems biology and genetic association testing methodologies to identify biologic pathways associated with neurocognitive and behavior outcomes after TBI. We then evaluated whether genes/single nucleotide polymorphism (SNPs) associated with these biologic pathways were more likely to demonstrate a relationship (i.e., enrichment) with short and long-term behavioral outcomes after early childhood TBI compared to genes/SNPs not associated with these biologic pathways.

**Setting:** Outpatient research setting.

**Participants:**140 children, ages 3–6:11 years at time of injury, admitted for a TBI or orthopedic injury (OI).

**Interventions:** Not Applicable.

**Main Outcome Measures:** Child behavior checklist total problems T score.

**Results:** Systems biology methodology identified neuronal systems and neurotransmitter signaling (Glutamate receptor, dopamine, serotonin, and calcium signaling), inflammatory response, cell death, immune systems, and brain development as important biologic pathways to neurocognitive and behavioral outcomes after TBI. At 6 months post injury, the group (TBI versus OI) by polymorphism interaction was significant when the aggregate signal from the highest ranked 40% of case gene associations was compared to the control set of genes. At ~ 7 years post injury, the selected polymorphisms had a significant main effect after controlling for injury type when the aggregate signal from the highest ranked 10% of the case genes were compared to the control set of genes

**Conclusions:** Findings demonstrate the promise of applying a genomics approach, informed by systems biology, to understanding behavioral recovery after pediatric TBI. A mixture of biologic pathways and processes are associated with behavioral recovery, specifically genes associated with cell death, inflammatory response, neurotransmitter signaling, and brain development. These results provide insights into the complex biology of TBI recovery.

## Background

Traumatic brain injury (TBI) is one of the most common causes of morbidity and mortality in children and young adults (Bruns and Hauser, 2003; Faul et al., 2010; Faul and Coronado, 2015; Popernack et al., 2015; Taylor et al., 2017). Behavior problems constitute a predominant aspect of post TBI morbidity (Arciniegas and Wortzel, 2014; Max, 2014; Babikian et al., 2015; Kennedy et al., 2017). Neurocognitive and behavioral issues, including attention problems, poor processing speed, and internalizing and externalizing behavioral problems, are among the most burdensome sequelae long-term after TBI and have a significant impact on everyday function (Babikian et al., 2015). Various injury-related, host, and environmental factors are linked to behavioral outcomes after injury (Babikian et al., 2015). However, a comprehensive biopsychosocial model of recovery is lacking, specifically an understanding of the role of genetic factors.

The role of genetics in behavioral recovery after TBI is incompletely understood. Research on the association of genetic factors with behavioral and cognitive outcomes after TBI is growing, in both adults and children (Diaz-Arrastia and Baxter, 2006; Jordan, 2007; Dardiotis et al., 2010; McAllister, 2010, 2015; Kurowski et al., 2012; Weaver et al., 2012; Davidson et al., 2014; Lipsky and Lin, 2015; Kassam et al., 2016). Prior work demonstrated associations of inflammatory, dopamine, neuroplasticity, cognitive, and cell metabolism-related genes and polymorphisms with outcomes after brain injury (Weaver et al., 2012; McAllister, 2015; Kurowski et al., 2017a). However, this prior work is primarily limited to narrow evaluations of a candidate gene and single polymorphism (Kurowski et al., 2017a). Based on the modest effect size of the associated variants, any one candidate gene or polymorphism is unlikely to explain substantial variation in recovery. Rather, many genes and variants are likely to contribute to recovery after brain injury (McAllister, 2015). Therefore, alternative approaches that consider multiple genes or polymorphisms are needed to characterize the role of genetic factors in outcomes after brain injury. Similarly, broader systems-level perspectives, rather than reductionist perspectives, that consider all elements of a particular system are needed for genetic studies (Ideker et al., 2001). Studies of genetic association for complex traits indicate that multiple biologic pathways are likely to underlie any given genetic association (Hirschhorn, 2005).

Within the field of TBI recovery, narrow genetic approaches focusing on a single variant at a time have predominated (Kurowski et al., 2012, 2017a). However, emerging research suggests that recovery after TBI is a complex trait and is influenced by multiple genes plus environmental factors (McAllister, 2015; Kurowski et al., 2017a,b; Treble-Barna et al., 2017c). As such, any one variant is likely to have a relatively modest effect given the probability that good or poor recovery reflects a complex interplay of many genes, each with modest effects. Recovery after TBI likely is influenced by a complex neurodevelopmental network of genes. An integrative, systems-biology based approach may help to improve the understanding of how a network of genes, rather than one or only a few genes, is associated with recovery. Such an approach also would provide novel insights into the biologic processes and networks that influence recovery (Kurowski et al., 2017a).

We hypothesized that the genes associated with behavioral recovery from TBI will be enriched for biologic pathways underpinning complex neurocognitive and behavioral networks. To test this hypothesis, we combined systems biology and traditional genetic association testing to identify biologic pathways or systems associated with early and later behavior outcomes after early childhood TBI. Based on a systematic review of primarily candidate gene association studies in the TBI literature and a systems biology informatics approach, we previously identified overrepresentation of gene variants in two primary biologic processes related to broad clinical outcomes following TBI: *response to injury* (cell proliferation, cell death, inflammatory response, cellular metabolism) and *neurocognitive and behavioral reserve* (brain development, cognition, and behavior) (Kurowski et al., 2017a). In the present study, we conducted a gene-enrichment analysis, in combination with systems biology methodology, of prospectively collected observational data. We hypothesized that single nucleotide polymorphisms associated with early (~6 months post injury) and long-term (~7 years post injury) behavioral outcomes would be enriched within genes belonging to neurocognitive and behavioral processes or pathways identified through use of systems biology methodology. We also expected to identify novel genes and polymorphisms likely important to these neurocognitive and behavioral processes that have not yet been evaluated in relation to TBI recovery.

## Methods

### Design

Cross-sectional evaluation of early and long-term behavioral outcomes in a longitudinal cohort of children with orthopedic injuries (OI) and TBIs.

### Participants

Children who sustained a TBI or OI from age 3 to age 6 years, 11 months were recruited from three tertiary care children's hospitals and one tertiary care, general hospital in Ohio (Yeates et al., 2010; Wade et al., 2016; Treble-Barna et al., 2017a). Inclusion criteria included hospitalization overnight for traumatic injury (TBI or OI), no evidence of child abuse as the cause of the injury, no history of documented neurological problems or developmental delays preinjury, and English as the primary language spoken in the home. Severity of TBI was characterized using the lowest post-resuscitation Glasgow Coma Scale (GCS) score (Teasdale and Jennett, 1974). GCS scores range from 3–15, with lower scores indication a more severe injury. Severe TBI was defined as a GCS score less than or equal to 8. Moderate TBI was defined as a GCS score of 9–12 with or without abnormal neuroimaging or a higher GCS score with abnormal neuroimaging as defined by an intracranial or parenchymal injury or depressed skull fracture. Mild TBI was defined as a GCS score ≥ 13 without abnormal neuroimaging. The OI group included children who sustained a bone fracture (not including skull fractures), had an overnight stay in the hospital, and did not exhibit alterations in consciousness or other signs or symptoms of head trauma or brain injury. The study was completed in accordance with Institutional Review Board (IRB) guidelines. Written informed consent was obtained in accordance with the Declaration of Helsinki. Written parental/guardian consent was obtained for all participants.

### Measures

Parents completed the age-appropriate form of the Child Behavior Checklist (CBCL) (Achenbach and Rescorla, 2001). The CBCL is a parent-report measure of child behavioral adjustment and possesses high test–retest reliability and criterion-related validity. We used the age- and sex-standardized Total Problems *T* score to assess child behavioral adjustment early (~6 months post injury) and long-term (~ 7 years post injury) after injury. The *T* score is normed for age and sex, with an average score of 50 and standard deviation of 10. Higher scores reflect poorer behavioral adjustment. The CBCL is a National Institute of Neurological Disorders and Stroke (NINDS) recommended common data elements for pediatric TBI and well-validated for the pediatric TBI population (McCauley et al., 2012).

### DNA Collection, Genotyping, and Quality Control

**Figure 1:**
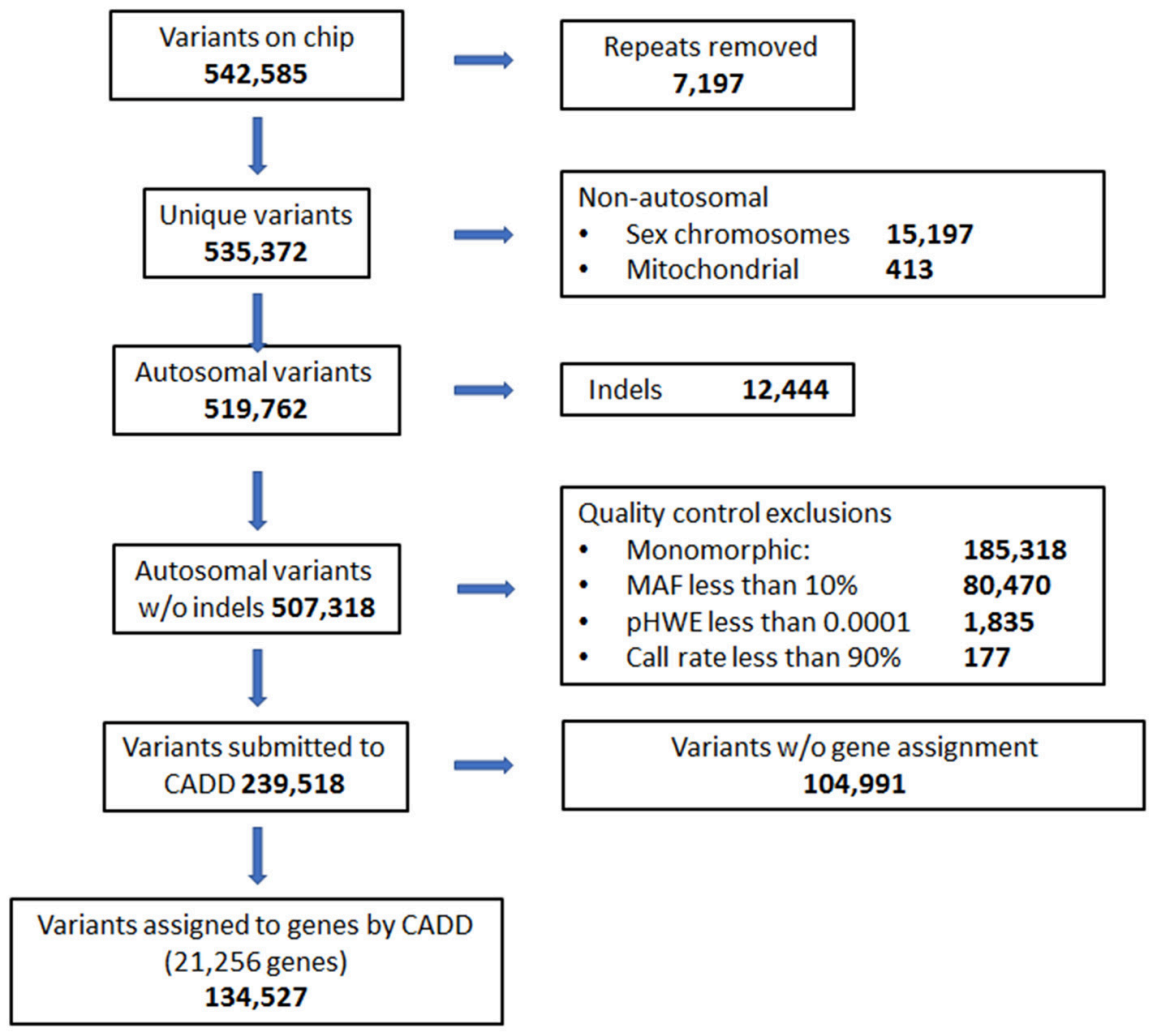
Quality control flow diagram and selection of genes and variants on exome chip to be used in analyses.

DNA was collected from saliva samples and purified using the Oragene OG-500 self-collection tubes (DNA Genotek, Ottawa, Canada) (Abraham et al., 2012). The HumanExome v1.1 Bead Chip (Illumina, San Diego, CA) was used to perform genotyping using the Illumina iScan. SNPs from the sex chromosome, mitochondrial, and indels were excluded from analysis. Quality of SNP calls from the chip were also evaluated. SNPs that failed Hardy Weinberg Equilibrium (p <0.0001) or had minor allele frequencies below 10% were excluded. Thresholds for quality control for call rates at individual and SNP levels were 99 and 90%, respectively. Combined Annotation Dependent Depletion (CADD) was used to annotate SNPs with respect to associated gene and type of variant (Kircher et al., 2014). SNPs located in intergenic regions and not associated with a specific gene according to CADD annotation were also excluded prior to analysis. The exome chip comprised 542,585 variants initially, and 134,527 variants remained after exclusions (Figure 1).

### Analyses Overview

We combined systems biology and genetic association testing methodologies to evaluate whether genes/SNPs associated with neurocognitive and behavior-related biologic pathways were more likely to demonstrate a relationship (i.e., enrichment) with short and long-term behavioral outcomes after early childhood TBI compared to genes/SNPs not associated with these biologic pathways.

### Demographic and Outcome Measure Comparison Between and Within Participants With and Without TBI

Sex, race, age at injury, age at short-term follow-up, age at long-term follow-up, zSES, GCS, CBCL total problems short-term and CBCL total problems long-term were compared between TBI and OI groups using an independent *t*-test for continuous variables or chi-squared test for categorical variables. Comparison between CBCL total problems short-term and CBCL total problems long-term within groups was completed using a paired *t*-test. A level of significance was established at alpha = 0.05.

### System Biology Platform

Information from a literature review was used to identify known TBI-associated genes (Kurowski et al., 2017a). Based on this review,18 genes were identified that previously showed an association with neurocognitive and behavioral outcomes after TBI:(Kurowski et al., 2017a) angiotensin I converting enzyme (ACE), adenosine A1 receptor (ADORA1), Ankyrin repeat and kinase domain contacting 1 (ANKK1), apolipoprotein E (APOE), BCL2, apoptosis regulator (BCL2), brain derived neurotrophic factor (BDNF), BMX non-receptor tyrosine kinase (BMX), catechol-O-methyltransferase (COMT), dopamine beta-hydroxylase (DBH), fatty acid amide hydrolase (FAAH), glutamate decarboxylase 1 (GAD1), glutamate ionotropic receptor NMDA type subunit 2A (GRIN2A), monoamine oxidase A (MAOA), NADH dehydrogenase, subunit 1 (MT-ND1), NADH dehydrogenase, subunit 3 (MT-ND3), neuroglobin (NGB), solute carrier family 6 member 4 (SLC6A4), WW and C2 domain containing 1 (WWC1). These 18 genes represented our known or “training” gene list. The training gene list was then evaluated using a systems biology platform, ToppGene Suite, to identify biologic pathways likely associated with brain injury recovery. Using these brain injury recovery and brain injury-related biologic pathways, additional genes, i.e., case genes (exclusive of the training set), likely to be highly associated with these brain injury recovery and brain injury-related biologic pathways were compiled. The case genes were ranked based on their functional similarity to the training set using the ToppGene application (ToppGene Suite) with default parameters (Chen et al., 2007). ToppGene Suite is a comprehensive platform used for gene set enrichment analyses and machine learning-based candidate gene ranking (Chen et al., 2009). Functional similarity among training and case genes was computed using a variety of gene annotations: pathways, biological processes, phenotype, literature, protein interactions, and co-expression.

### Control Gene Set Identification

To identify a control set of genes, i.e., genes not related to TBI outcomes or associated with biologic systems or pathways implicated in TBI, we used results from the ToppGene Suite analysis described above to identify genes unlikely to be related to TBI outcomes or brain injury-related biologic processes.

### Genetic Association Analyses

Analyses were conducted using PLINK v.07 (http://pngu.mgh.harvard.edu/purcell/plink) (Purcell et al., 2007). Prior to analyses, child outcome data were reviewed and reduced according to the following rules to limit the potential influence of outliers: (1) participants with changes in outcome scores between the short-term and long-term time point that exceeded three standard deviations of change were excluded from respective analyses (*n* = 0); and (2) outcome scores of T > 90 (4 standard deviations above the normative mean) were Winsorized to 90 (*n* = 1) (Treble-Barna et al., 2017c). Prior to genetic analyses, cryptic relatedness (i.e., genetic similarly between individuals indicating unexpected relationship) was checked using Graphical Representation of Relationship (GRR) (Abecasis et al., 2001). Principal component analysis was employed to confirm European and African continental ancestry using 482 validated ancestry informative markers (Tandon et al., 2011). Concordance with self-reported race was > 95%.

To identify significant SNPs, we used linear models for association of each SNP as well as SNP X injury group interaction (to test whether a SNP's association with outcome differed by injury group). In all association tests, we used an additive genetic model where major homozygotes were coded as 0, heterozygotes as 1, and minor homozygotes as 2. Covariates initially included the child's pre-injury functioning on the CBCL (i.e., retrospective rating of child's behavior prior to injury) and socioeconomic status (defined by averaging sample z scores for maternal education and median census track income) (Yeates et al., 2010) and were then trimmed if non-significant. The first principal component from the ancestry analysis was included to correct for population structure as it captured the vast majority of variation. PLINK v.07 was used for association tests.

### Gene Enrichment Analyses

Gene enrichment analyses were performed using R software (http://www.R-project.org). Enrichment is indicated when a greater number of genetic associations are present in case vs. control genes. To test for enrichment, we compared the number of associations in our case set that met the *p* < 0.05 threshold to the number of associations meeting the same criteria in over 10,000 matched runs of our control set of genes. Given the large number of case genes, we used the ranking from the systems biology approach to create subsets of the larger set of ranked genes in order to determine if a more restrictive list was sufficient. We created subsets gene lists and the full gene list. These lists include 0% (training set only), 5% (training set plus top 5% of ranked genes), 10% (training set plus 10% of ranked genes of ranked genes), etc., until all genes are included, thus these lists are described as percentiles of case genes. We then selected sets of control genes (for each set of case genes, 10,000 control gene were selected for each set). SNPs for the control set were matched to the case gene set on the ratio of minor allele frequency (MAF) using MAF bands as follows: 10–15%; 15–20%:%; 20–30%:%; 30–50%. We then tested whether our outcomes were associated with polymorphisms within our case and control genes. Using the 10,000 matched runs from the control sets, we established the 95th percentile for the number of associations expected by chance. When the number of nominal (*p* < 0.05) associations in the case genes exceeded the 95th percentile expected by chance, the case genes at these percentile epochs were considered to be enriched for genes/polymorphisms associated with outcomes. While we recognize that many of the nominally associated genes are false positive association due to an inflated family-wise error rate, our question is whether there is enrichment of association across the overall set of variants (rather than any specific variant). The 10,000 matched control runs (for each comparison group) ensures that the *p*-value accounts for the number of variants tested. We did not correct for multiple testing among the subsets of variants as these are not mutually exclusive subgroups.

## Results

### Participants

Of the 221 participants enrolled in the original study, 140 provided DNA samples and had covariate and outcome data available (Table 1). Participants with genetic data did not differ significantly from those without genetic data in demographic characteristics or on various study measures. Of those included in the present analyses, 10 had mild TBI, 43 had moderate TBI, 16 had severe TBI, and 71 had OI. The TBI and OI groups did not differ significantly in race, sex, age at injury, age at assessment, or socioeconomic status (Table 1). Children with TBI had poorer behavioral adjustment than children with OI at both the short-term and long-term assessments (Table 1). The CBCL total problems short-term and long-term were similar within the OI group (*p* = 0.7959), but increased at long-term time point within the TBI group (*p* = 0.026). These results are consistent with our prior reports based on the larger sample from this cohort (Narad et al., 2016; Treble-Barna et al., 2017b).

**Table 1:** Demographics: participant characteristics by injury group.

	**OI**	**TBI**	***P***
	***N* = 71**	***N* = 69**	
Gender, *n* (%)			0.601
Male	37 (52.1)	39 (56.5)	
Female	34 (47.9)	30 (43.5)	
Race, *n* (%)			0.289
White	55 (77.5)	48 (69.6)	
Non-white	16 (22.5)	21 (30.4)	
Age at injury in years, M (SD)	5.11 (1.06)	5.10 (1.15)	0.927
zSES, M (SD)	0.11 (0.95)	−0.14 (0.99)	0.140
GCS, M (SD)	NA	11.23 (4.45)	NA
CBCL Total Problems at injury, M (SD)	45.62 (11.80)	50.43. (13.63)	0.027
CBCL Total Problems short-term, M (SD)	43.76 (9.22)	52.80. (13.86)	<0.001
CBCL Total Problems long-term, M (SD)	45.12 (10.87)	55.79 (12.96)	<0.001

*CBCL, Child Behavior Checklist; GCS, Glasgow Coma Scale; zSES, socioeconomic status z score*.

### Systems Biology Approach

**Figure 2:**
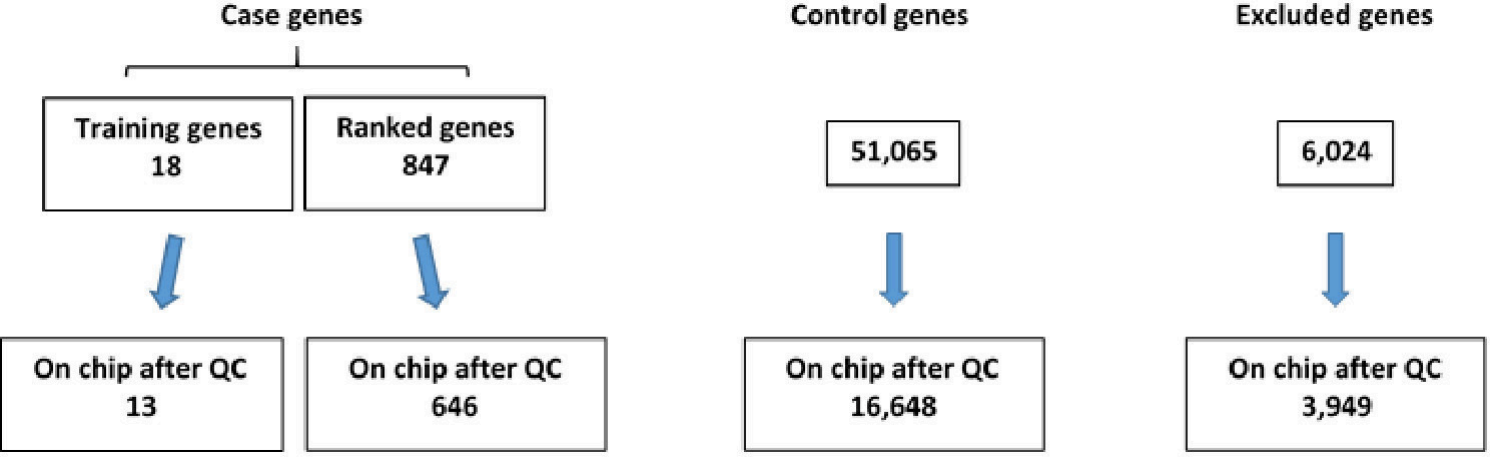
Number of case and control genes and excluded genes on exome chip after quality control (QC) was completed.

After completion of genotyping and initial quality control, 13 of the 18 original “training” set genes and 646 of 847 compiled case genes (as described in Methods) were found to be represented on the exome chip (Figure 2). Further, 16,648 control genes were identified on the exome chip and 3,949 genes were excluded as they demonstrated a potential relationship with TBI recovery or TBI-related biologic processes, thus not fitting the criteria for a case or control gene (Figure 2).

**Figure 3:**
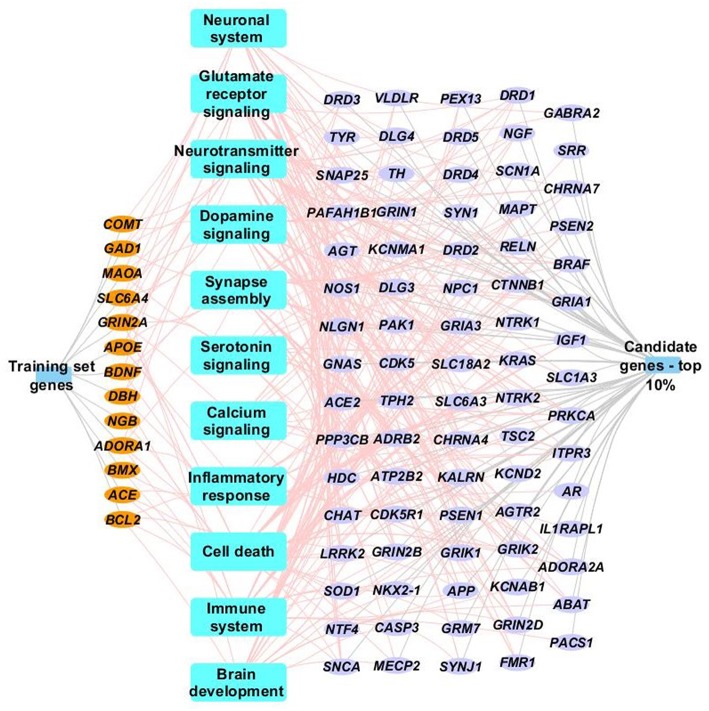
Network representation of select enriched biological processes, pathways and genes. Select enriched biological processes and pathways (*p* <0.05) are represented as blue rectangles along with their associated training set genes (orange ellipse) and top 10% case genes (purple ellipse). Only genes (training or case sets) that are associated with terms identified by ToppGene suite as being influential to brain injury recovery are shown in the network. Complete details of the functional enrichment results can be found in Supplementary Table.

The 18 “training” genes and the ToppGene-ranked top 10% of case genes (85/847) were also combined and functional enrichment analysis was performed to characterize key biological processes. Figure 3 depicts a network representation of selected biologic processes identified as important to TBI neurocognitive and behavioral recovery using ToppGene Suite. Enriched biological processes and pathways included neuronal systems and neurotransmitter signaling (Glutamate receptor, dopamine, serotonin, and calcium signaling), inflammatory response, cell death, immune systems, and brain development (see Figure 3 and Supplementary File for a complete list of enriched biological processes and pathways).

### Gene-Enrichment Analysis Results

**Figure 4:**
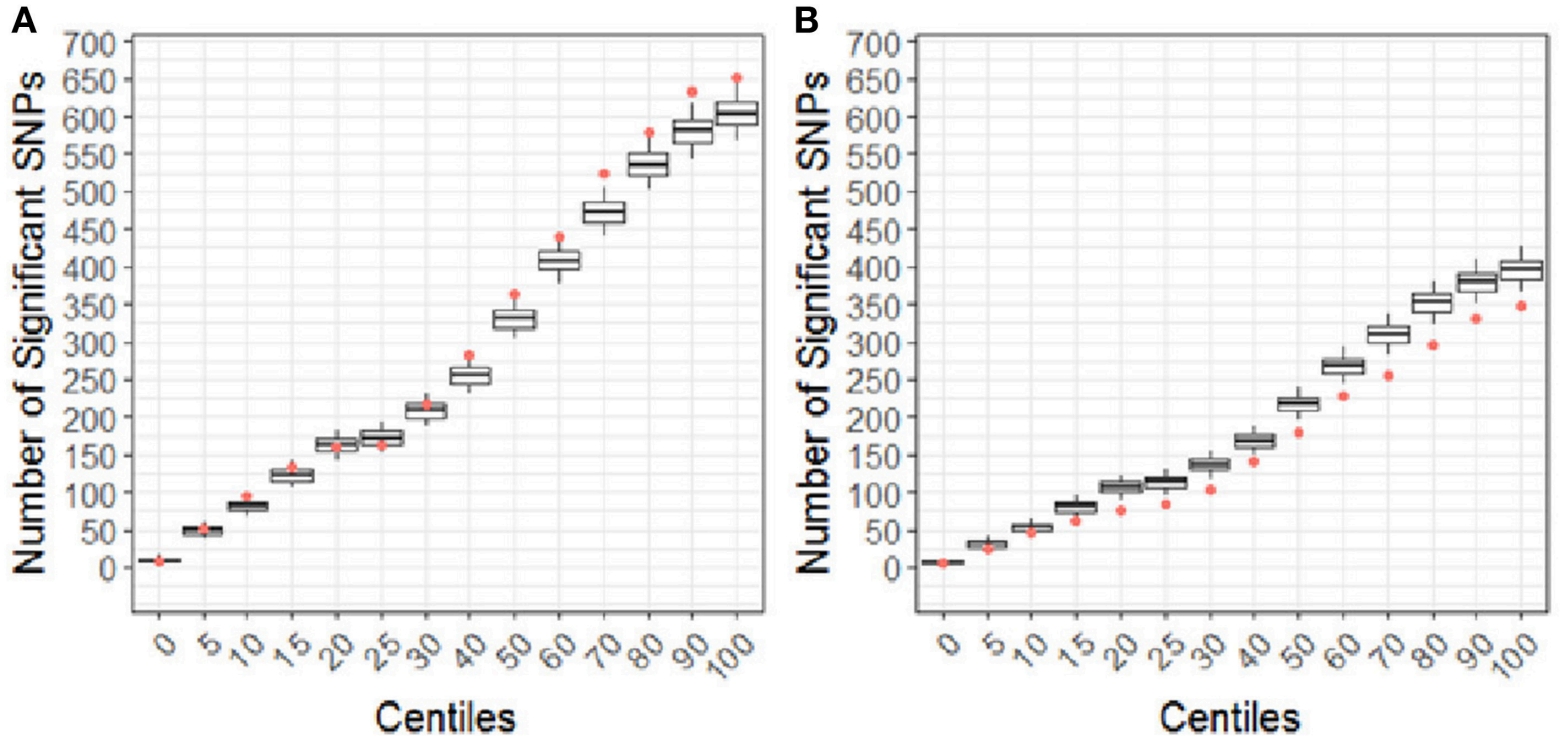
Differential response to outcome by injury type (TBI vs. OI) for short **(A)** and long **(B)** term of behavioral outcomes. Centiles represent the portion of case genes used in the genetic association analysis: 0% includes that only the training list was included, 5th percentile includes the training list plus the top 5% highest ranked genes, 10th centile includes the training list plus the top 10% of ranked genes and so forth until all ranked genes were included (i.e., 100th centile). Vertical axis represents the number of single nucleotide polymorphisms. Box plots represent the number of significant SNPs in the 10,000 runs of control gene SNPs. The dot indicates that number of nominal associations (*p* < 0.05) identified in case genes. Enrichment is indicated when a greater number of genetic associations are present in case vs. control genes; therefore, when the number of associations in case genes (represented by the dot) exceeded the upper 95th percentile threshold in the run of control genes. In panel A, at the 40th centile and above, the dot is above the 95th percentile indicating that there are more case-gene SNPs significantly associated with short-term behavioral outcomes than what would be expected by chance, indicating enrichment. In panel B, the number of case gene SNP associations are below number of associations in the control set, indicating that case genes are unlikely to be enriched for pathways specific to long-term behavioral outcomes after TBI compared to OI. Because this figure represents the point estimates for the interaction term of group (TBI vs. OI) with SNPs, these findings demonstrate that there is differential outcomes in the TBI vs. the OI group in the short-term rather than the long-term.

**Figure 5:**
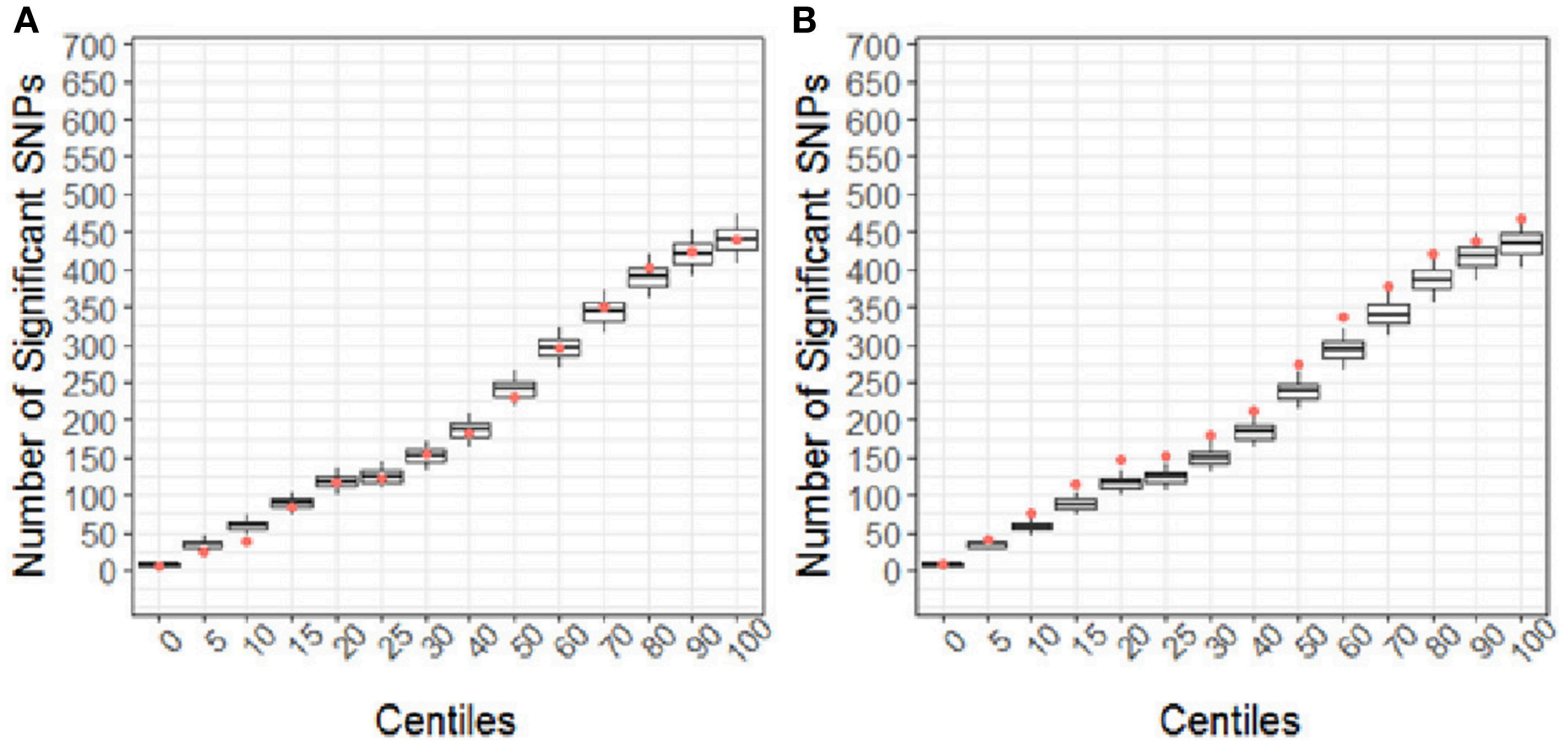
Short **(A)** and Long-term behavioral outcomes **(B)** when controlling for injury type (TBI vs. OI). Centiles represent the portion of case genes used in the genetic association analysis: 0% includes that only the training list was included, 5th percentile includes the training list plus the top 5% highest ranked genes, 10th centile includes the training list plus the top 10% of ranked genes and so forth until all ranked genes were included (i.e., 100th centile). Vertical axis represents the number of single nucleotide polymorphisms. Box plots represent the number of significant SNPs in the 10,000 runs of control gene SNPs. The dot indicates that number of nominal associations (*p* < 0.05) identified in case genes. Enrichment is indicated when a greater number of genetic associations are present in case vs. control genes; therefore, when the number of associations in case genes (represented by the dot) exceeded the upper 95th percentile threshold in the run of control genes. In panel A, no enrichment is identified for short-term outcomes. In panel B, at the 10th to the 60th centile, there are more case-gene SNPs associated with long-term behavioral outcomes than what would be expected by chance. Because this figure demonstrates main effects controlling for group (TBI vs. OI), these findings indicate that a complex network of genes/polymorphisms is associated with long-term behavioral outcomes after traumatic injury of any sort (i.e., TBI or OI).

Using the CBCL Total Problems score as the dependent variable, the number of nominally associated SNPs in the case genes were compared to number of associations expected to be identified by chance in the control set of genes. At the early time point (~ 6 months post injury), the group (TBI vs. OI) by polymorphism interaction was significant. At the 40th percentile and higher centiles in the case set of genes, the number of SNPs associated with the CBCL Total Problems score was larger than what was expected by chance (Figure 4, *p* range:0.004–0.043), indicating that the aggregate association of case genes/polymorphisms is larger compared to the control genes/polymorphisms and more likely to be associated with differential behavioral outcomes in those with TBI compared to OI over the first 6 months post injury. At the long-term follow-up (~ 7 years post-injury), the group (TBI vs. OI) by gene/polymorphism interaction was not significant. This result indicates that it is unlikely enrichment is present in case genes when evaluating whether the case genes are associated with differential long-term behavioral outcomes between the TBI and OI groups. However, the main effect for SNPs was significantly associated with long-term behavioral outcomes after controlling for injury type (TBI vs. OI). At the 10th percentile and higher centiles, the number of SNPs associated with the CBCL Total Problems score was larger in the case genes compared what is expected by chance (Figure 5, *p* range: 0.001 to 0.033), indicating that the network of genes/polymorphisms in the case genes is associated with differential long-term behavioral outcomes after traumatic injury (i.e., for both TBI and OI).

## Discussion

The results of this study suggest that behavioral outcomes after early childhood TBI are associated with a combination of genes underpinning an array of biologic processes, including immune system functioning, inflammatory response, cell death, neurotransmitter signaling (serotonin, dopamine, glutamate receptor, and calcium signaling), and brain development. These results indicate that a complex relationship exists among a variety of genes, the biologic process they influence, and behavioral recovery after early childhood TBI. The findings suggest that an aggregate signal from a set of genes may provide important insights to genetic influences on behavioral outcomes of pediatric TBI.

It is likely that certain genetic factors will be differentially associated with earlier and later outcomes after injury. Our results indicate that the network of neurocognitive and behavioral related genes evaluated in this work may be specific to shorter-term behavioral outcomes after pediatric TBI. Later after injury, genetic influences on behavioral outcomes did not differ between TBI and OI groups although a network of genes was associated with long-term behavioral outcomes regardless of injury type (TBI or OI). This finding indicates that the network of genes evaluated might be specific to shorter-term recovery after TBI and long-term behavioral outcomes after any of sort of trauma in general or may contribute to variations in behavior in the population at large.

In agreement with the initial hypotheses, several genes that have not previously been studied in TBI populations were identified, indicating that a broader focus on the genetic influences upon biologic processes or pathways might assist in elucidating the aggregate genetic factors associated with recovery after TBI and supports the concept that any one gene is unlikely to be as important as a combination of genes. These findings are in agreement with prior work that indicates the biology of behavioral and psychiatric traits is incompletely understood and most of the genes involved in behaviorally related traits are difficult to predict *a priori* (Gelernter, 2015). A better understanding of the systems or processes associated with recovery from TBI may help to inform prognosis and development of treatments that target a variety of biologic and physiologic pathways. Overall, this early work demonstrates the promise of applying a genomics approach, informed by systems biology, to understanding behavioral recovery in the short- and long-term after pediatric TBI.

Our results suggest that behavioral recovery from TBI is a complex trait. Complex trait is a term that refers to traits that do not typically follow Mendelian inheritance patterns (e.g., dominant, recessive, or sex-linked inheritance) (Manolio et al., 2009; Gelernter, 2015). Complex inheritance patterns of behavioral traits may be related to various factors, including the presence of multiple risk alleles; gene-gene interactions; epigenetic effects; and gene-environment interactions (Bookman et al., 2011; Gelernter, 2015). Although not the focus of this study, integrating broader consideration of how genes interact across biological systems and with the environment will be critical to understanding the complex nature of neurocognitive and behavioral recovery after TBI.

A potential next step is to understand when certain biologic processes are most critical in recovery after brain injury. That is, linking gene profiles of biologic processes associated with recovery with specific gene expression and proteomic factors over time after injury (acute to chronic) would assist in characterizing when these biologic processes are most critical for recovery. For example, inflammatory processes may be critical acutely and chronically after injury (Dalla Libera et al., 2011; White et al., 2013; Juengst et al., 2015; Kumar et al., 2015; Witcher et al., 2015; McKee and Lukens, 2016; Lagraoui et al., 2017), whereas neurotransmitters and behavioral signaling may be more important primarily later after injury (McAllister, 2009; Willmott et al., 2013; Failla et al., 2015; Myrga et al., 2015, 2016; Kurowski et al., 2017a,b; Treble-Barna et al., 2017c). Characterizing these processes would be an important step toward determining when specific treatments may have the greatest impact on recovery and could also severe as a biomarker to provide a more proximal measure of treatment benefits. Ultimately, optimal brain injury treatment may require targeting multiple biologic processes at various times post injury to maximize recovery.

Further, recovery after brain injury is associated with a complex interaction among cellular, host, treatment, and environmental factors (Adams et al., 2017; Kenzie et al., 2017, 2018). Thus, to move toward a true precision medicine approach, an improved understanding how genetic and other biologic factors interact with other host, injury-related, treatment, and environmental factors to influence recovery will be critical. Age at injury, sex, and environmental factors, such as parenting style, may interact with genetic factors to influence recovery (Treble-Barna et al., 2016; Kurowski et al., 2017b; Smith-Paine et al., 2018). Prior work by our group demonstrated that, in an adverse parenting environment, a catechol-o-methyltransferase (COMT) polymorphism is protective in regards to neurocognitive functioning 18 months after pediatric TBI (Kurowski et al., 2017b) Integrating genetics, biologic, psychosocial, environmental, and individual factors into analyses to inform development of a comprehensive biopsychosocial model of recovery would allow for better development of individualized prognosis and treatment plans, but requires large-scale, collaborative, multicenter studies (Cotter et al., 2017; Kenzie et al., 2017).

### Limitations and Considerations for Future Studies

Several limitations should be considered when interpreting the results from this study. Previous research was used in defining the case set of genes; therefore, selection bias related to publication bias of genetic reports may influence the results. Other influential genes may not have been considered. This study also lacks a validation/replication cohort and replication of these findings should be a focus of future research. An exome chip was utilized in this study, and the genes and polymorphisms evaluated were restricted to those polymorphisms represented on the chip. Although difficult in practical terms, a GWAS approach may help elucidate additional genes and pathways that may have been missed. Imputation was also considered; however, confidence in obtaining valid and reliable imputation was low due the sparseness of the data available on the exome chip. Additionally, since we were interested in evidence of overall gene enrichment, rather than specific associations, imputation is likely unwarranted. The study also focused on children with mild to severe injuries; however, due to overall limited sample sizes, subgroup analyses are not justified. Future studies should consider how injury severity may or may not moderate genetic association. Furthermore, limited sample sizes also precluded consideration of other modifying factors, such as the family environment. This study also focused on children injured at an early age; therefore, generalization to other age groups should be done cautiously. The outcome utilized represents parent/proxy report of behavior; therefore, the association with self-report or other objective measures of behavioral functioning are uncertain. Future studies should also consider applying similar methodology to identify genetic risks for specific behavioral subtypes and other outcomes as well.

## Conclusion

Recovery after TBI is complex, and multiple biologic processes likely influence outcomes. Broader approaches that consider a system or network of genes provides a perspective on how genes in combination influence behavioral recovery after TBI. Given what is known about the processes involved in recovery, this broader approach may more closely align with the underlying physiologic changes than narrow approaches that focus on single genes in isolation. Future research is needed to consider the combined influence of genetic, host, injury-related, and environmental factors to move toward precision medicine approaches for TBI recovery in children.

## Author Contributions

All authors listed have made a substantial, direct and intellectual contribution to the work, and approved it for publication.

### Conflict of Interest Statement

The authors declare that the research was conducted in the absence of any commercial or financial relationships that could be construed as a potential conflict of interest.

## References

[B1] Abecasis G. R. Cherny S. S. Cookson W. O. Cardon L. R. (2001). GRR: graphical representation of relationship errors. Bioinformat. Appl. Note 17, 742–743. 10.1093/bioinformatics/17.8.742 11524377

[B2] Abraham J. E. Maranian M. J. Spiteri I. Russell R. Ingle S. Luccarini C.. (2012). Saliva samples are a viable alternative to blood samples as a source of DNA for high throughput genotyping. BMC Med. Genom. 5:19. 10.1186/1755-8794-5-19 22647440 PMC3497576

[B3] Achenbach T. M. Rescorla L. A. (2001). Manual for ASEBA School-Age Forms & Profiles. Burlington, VT: University of Vermont, Research Center for Children, Youth, & Families.

[B4] Adams S. M. Conley Y. P. Wagner A. K. Jha R. M. Clark R. S. Poloyac S. M.. (2017). The pharmacogenomics of severe traumatic brain injury. Pharmacogenomics 18, 1413–1425. 10.2217/pgs-2017-0073 28975867 PMC5694019

[B5] Arciniegas D. B. Wortzel H. S. (2014). Emotional and behavioral dyscontrol after traumatic brain injury. Psychiatr. Clin. North Am. 37, 31–53. 10.1016/j.psc.2013.12.001 24529422

[B6] Babikian T. Merkley T. Savage R. C. Giza C. C. Levin H. (2015). Chronic aspects of pediatric traumatic brain injury: review of the literature. J. Neurotrauma. 32, 1849–1860. 10.1089/neu.2015.3971 26414654

[B7] Bookman E. B. McAllister K. Gillanders E. Wanke K. Balshaw D. Rutter J.. (2011). Gene-environment interplay in common complex diseases: forging an integrative model-recommendations from an NIH workshop. Genet. Epidemiol. 35, 217–25. 10.1002/gepi.20571 21308768 PMC3228883

[B8] Bruns J. Jr. Hauser W. A. (2003). The epidemiology of traumatic brain injury: a review. Epilepsia 44 (Suppl 10), 2–10. 10.1046/j.1528-1157.44.s10.3.x 14511388

[B9] Chen J. Bardes E. E. Aronow B. J. Jegga A. G. (2009). ToppGene Suite for gene list enrichment analysis and candidate gene prioritization. Nucleic Acids Res. 37, W305–311. 10.1093/nar/gkp427 19465376 PMC2703978

[B10] Chen J. Xu H. Aronow B. J. Jegga A. G. (2007). Improved human disease candidate gene prioritization using mouse phenotype. BMC Bioinformat. 8:392. 10.1186/1471-2105-8-392 17939863 PMC2194797

[B11] Cotter D. Kelso A. Neligan A. (2017). Genetic biomarkers of posttraumatic epilepsy: a systematic review. Seizure 46, 53–58. 10.1016/j.seizure.2017.02.002 28242442

[B12] Dalla Libera A. L. Regner A. de Paoli J. Centenaro L. Martins T. T. Simon D. (2011). IL-6 polymorphism associated with fatal outcome in patients with severe traumatic brain injury. Brain Inj. 25, 365–369. 10.3109/02699052.2011.556107 21314275

[B13] Dardiotis E. Fountas K. N. Dardioti M. Xiromerisiou G. Kapsalaki E. Tasiou A.. (2010). Genetic association studies in patients with traumatic brain injury. Neurosurg. Focus. 28:E9. 10.3171/2009.10.FOCUS09215 20043724

[B14] Davidson J. Cusimano M. D. Bendena W. G. (2014). Post-traumatic brain injury: genetic susceptibility to outcome. Neuroscientist. 21, 424–441. 10.1177/1073858414543150 25059577

[B15] Diaz-Arrastia R. Baxter V. K. (2006). Genetic factors in outcome after traumatic brain injury: what the human genome project can teach us about brain trauma. J. Head Trauma. Rehabil. 21, 361–374. 10.1097/00001199-200607000-00007 16915011

[B16] Failla M. D. Myrga J. M. Ricker J. H. Dixon C. E. Conley Y. P. Wagner A. K. (2015). Posttraumatic brain injury cognitive performance is moderated by variation within ANKK1 and DRD2 Genes. J. Head Trauma. Rehabil. 30, E54–66. 10.1097/HTR.0000000000000118 25931179 PMC4626432

[B17] Faul M. Coronado V. (2015). Epidemiology of traumatic brain injury. *Handb Clin*. Neurol. 127, 3–13. 10.1016/B978-0-444-52892-6.00001-5 25702206

[B18] Faul M. Xu L. Wald M. Coronado V. (2010). Traumatic brain injury in the United States: emergency department visits, hospitalizations and deaths 2002–2006, in: Centers for Disease Control and Prevention, National Center for Injury Prevention and Contro (Atlanta, GA). Available online at: http://www.cdc.gov/traumaticbraininjury/pdf/blue_book.pdf (accessed September 9, 2013). 10.15620/cdc.5571

[B19] Gelernter J. (2015). Genetics of complex traits in psychiatry. Biol Psychiatry, 77, 36–42. 10.1016/j.biopsych.2014.08.005 25444161 PMC4282183

[B20] Hirschhorn J. N. (2005). Genetic approaches to studying common diseases and complex traits. Pediatr. Res. 57(5 Pt 2), 74R−77R. 10.1203/01.PDR.0000159574.98964.87 15817501

[B21] Ideker T. Galitski T. Hood L. (2001). A new approach to decoding life: systems biology. Annu. Rev. Genomics Hum. Genet. 2, 343–372. 10.1146/annurev.genom.2.1.343 11701654

[B22] Jordan B. D. (2007). Genetic influences on outcome following traumatic brain injury. Neurochem. Res. 32, 905–915. 10.1007/s11064-006-9251-3 17342413

[B23] Juengst S. B. Kumar R. G. Failla M. D. Goyal A. Wagner A. K. (2015). Acute inflammatory biomarker profiles predict depression risk following moderate to severe traumatic brain injury. J. Head Trauma. Rehabil. 30, 207–218. 10.1097/HTR.0000000000000031 24590155

[B24] Kassam I. Gagnon F. Cusimano M. D. (2016). Association of the APOE-epsilon4 allele with outcome of traumatic brain injury in children and youth: a meta-analysis and meta-regression. J. Neurol. Neurosurg. Psychiatry. 87, 433–440. 10.1136/jnnp-2015-310500 25904811

[B25] Kennedy E. Cohen M. Munafò M. (2017). Childhood traumatic brain injury and the associations with risk behavior in adolescence and young adulthood: a systematic review. J. Head Trauma. Rehabil. 32, 425–432. 10.1097/HTR.0000000000000289 28092286 PMC5690295

[B26] Kenzie E. S. Parks E. L. Bigler E. D. Lim M. M. Chesnutt J. C. Wakeland W. (2017). Concussion as a multi-scale complex system: an interdisciplinary synthesis of current knowledge. Front. Neurol. 8:513. 10.3389/fneur.2017.00513 29033888 PMC5626937

[B27] Kenzie E. S. Parks E. L. Bigler E. D. Wright D. W. Lim M. M. Chesnutt J. C.. (2018). The dynamics of concussion: mapping pathophysiology, persistence, and recovery with causal-loop diagramming. Front. Neurol. 9:203. 10.3389/fneur.2018.00203 29670568 PMC5893805

[B28] Kircher M. Witten D. M. Jain P. O'Roak B. J. Cooper G. M. Shendure J. (2014). A general framework for estimating the relative pathogenicity of human genetic variants. Nat. Genet. 46, 310–315. 10.1038/ng.2892 24487276 PMC3992975

[B29] Kumar R. G. Boles J. A. Wagner A. K. (2015). Chronic inflammation after severe traumatic brain injury: characterization and associations with outcome at 6 and 12 months postinjury. J. Head Trauma Rehabil. 30, 369–381. 10.1097/HTR.0000000000000067 24901329

[B30] Kurowski B. Martin L. J. Wade S. L. (2012). Genetics and outcomes after traumatic brain injury (TBI): What do we know about pediatric TBI?. J. Pediatr. Rehabil. Med. 5, 217–231. 10.3233/PRM-2012-0214 23023254 PMC3625371

[B31] Kurowski B. G. Treble-Barna A. Pitzer A. J. Wade S. L. Martin L. J. Chima R. S.. (2017a). Applying systems biology methodology to identify genetic factors possibly associated with recovery after traumatic brain injury. J. Neurotrauma. 34, 2280–2290. 10.1089/neu.2016.4856 28301983 PMC5510694

[B32] Kurowski B. G. Treble-Barna A. Zang H. Zhang N. Martin L. J. Yeates K. O.. (2017b). Catechol-O-methyltransferase genotypes and parenting influence on long-term executive functioning after moderate to severe early childhood traumatic brain injury: an exploratory study. J. Head Trauma Rehabil. 32, 404–412. 10.1097/HTR.0000000000000281 28060209 PMC5498281

[B33] Lagraoui M. Sukumar G. Latoche J. R. Maynard S. K. Dalgard C. L. Schaefer B. C. (2017). Salsalate treatment following traumatic brain injury reduces inflammation and promotes a neuroprotective and neurogenic transcriptional response with concomitant functional recovery. Brain Behav. Immun. 61, 96–109. 10.1016/j.bbi.2016.12.005 27939247 PMC5316369

[B34] Lipsky R. H. Lin M. (2015). Genetic predictors of outcome following traumatic brain injury. Handb. Clin. Neurol. 127, 23–41. 10.1016/B978-0-444-52892-6.00003-9 25702208

[B35] Manolio T. A. Collins F. S. Cox N. J. Goldstein D. B. Hindorff L. A. Hunter D. J.. (2009). Finding the missing heritability of complex diseases. Nature. 461, 747–753. 10.1038/nature08494 19812666 PMC2831613

[B36] Max J. E. (2014). Neuropsychiatry of pediatric traumatic brain injury. Psychiatr. Clin. North Am. 37, 125–140. 10.1016/j.psc.2013.11.003 24529428 PMC3977029

[B37] McAllister T. W. (2009). Polymorphisms in genes modulating the dopamine system: do they inf luence outcome and response to medication after traumatic brain injury? J. Head Trauma Rehabil. 24, 65–68. 10.1097/HTR.0b013e3181996e6b 19158598 PMC3169998

[B38] McAllister T. W. (2010). Genetic factors modulating outcome after neurotrauma. PM&R. 2(12, Supplement 2), S241– 252. 10.1016/j.pmrj.2010.10.005 21172686

[B39] McAllister T. W. (2015). Genetic factors in traumatic brain injury. Handb. Clin. Neurol. 128, 723–739. 10.1016/B978-0-444-63521-1.00045-5 25701917

[B40] McCauley S. R. Wilde E. A. Anderson V. A. Bedell G. Beers S. R. Campbell T. F.. (2012). Recommendations for the use of common outcome measures in pediatric traumatic brain injury research. J. Neurotrauma. 29, 678–705. 10.1089/neu.2011.1838 21644810 PMC3289848

[B41] McKee C. A. Lukens J. R. (2016). Emerging roles for the immune system in traumatic brain injury. Front. Immunol. 7:556. 10.3389/fimmu.2016.00556 27994591 PMC5137185

[B42] Myrga J. M. Failla M. D. Ricker J. H. Dixon C. E. Conley Y. P. Arenth P. M.. (2015). A Dopamine pathway gene risk score for cognitive recovery following traumatic brain injury: methodological considerations, preliminary findings, and interactions with sex. J. Head Trauma Rehabil. 31, E15–29 10.1097/HTR.0000000000000199 26580694

[B43] Myrga J. M. Juengst S. B. Failla M. D. Conley Y. P. Arenth P. M. Grace A. A.. (2016). COMT and ANKK1 Genetics interact with depression to influence behavior following severe TBI: an initial assessment. Neurorehabilit. Neural Repair. 30, 920–930. 10.1177/1545968316648409 27154305 PMC5048493

[B44] Narad M. E. Treble-Barna A. Peugh J. Yeates K. O. Taylor H. G. Stancin T.. (2016). Recovery trajectories of executive functioning after pediatric TBI: a latent class growth modeling analysis. J. Head Trauma Rehabilit. 32, 98–106. 10.1097/HTR.0000000000000247 27455434 PMC5580343

[B45] Popernack M. L. Gray N. Reuter-Rice K. (2015). Moderate-to-severe traumatic brain injury in children: complications and rehabilitation strategies. J. Pediatr. Health Care. 29:e1–7. 10.1016/j.pedhc.2014.09.003 25449002 PMC4409446

[B46] Purcell S. Neale B. Todd-Brown K. Thomas L. Ferreira M. A. Bender D.. (2007). PLINK: a tool set for whole-genome association and population-based linkage analyses. Am. J. Hum. Genet. 81, 559–575. 10.1086/519795 17701901 PMC1950838

[B47] Smith-Paine J. Wade S. L. Treble-Barna A. Zhang N. Zang H. Martin L. J.. (2018). The moderating effect of the ankyrin repeat and kinase domain containing one gene on the association of family environment with longitudinal executive function following traumatic brain injury in early childhood: a preliminary study. J Neurotrauma. 35, 2796–2280. 10.1089/neu.2017.5388 29717626 PMC6247983

[B48] Tandon A. Patterson N. Reich D. (2011). Ancestry informative marker panels for African Americans based on subsets of commercially available SNP arrays. Genet Epidemiol. 35, 80–83. 10.1002/gepi.20550 21181899 PMC4386999

[B49] Taylor C. A. Bell J. M. Breiding M. J. Xu L. (2017). Traumatic brain injury–related emergency department visits, hospitalizations, and deaths — United States, 2007 and 2013. MMWR Surveill. Summ. 66, 1–18. 10.15585/mmwr.ss6609a1 28301451 PMC5829835

[B50] Teasdale G. Jennett B. (1974). Assessment of coma and impaired consciousness. A practical scale. Lancet 2, 81–84. 10.1016/S0140-6736(74)91639-0 4136544

[B51] Treble-Barna A. Schultz H. Minich N. Taylor H. G. Yeates K. O. Stancin T.. (2017b). Long-term classroom functioning and its association with neuropsychological and academic performance following traumatic brain injury during early childhood. Neuropsychology 31, 486–498. 10.1037/neu0000325 28627915 PMC5502733

[B52] Treble-Barna A. Wade S. L. Martin L. J. Pilipenko V. Yeates K. O. Taylor H. G.. (2017c). Influence of dopamine-related genes on neurobehavioral recovery after traumatic brain injury during early childhood. J. Neurotraum. 34, 1919–1931. 10.1089/neu.2016.4840 28323555 PMC5455258

[B53] Treble-Barna A. Zang H. Zhang N. Martin L. J. Yeates K. O. Taylor H. G.. (2016). Does apolipoprotein e4 status moderate the association of family environment with long-term child functioning following early moderate to severe traumatic brain injury? a preliminary study. J. Int. Neuropsychol. Soc. 22, 859–864. 10.1017/S1355617716000631 27480909 PMC5476473

[B54] Treble-Barna A. Zang H. Zhang N. Taylor G. Yeates K. O. Wade S. (2017a). Profile analysis of long-term neuropsychological functioning following traumatic brain injury in early Childhood. J Neurotrauma. 34, 353–362. 10.1089/neu.2016.4476 27080734 PMC5220574

[B55] Wade S. L. Zhang N. Yeates K. O. Stancin T. Taylor H. G. (2016). Social environmental moderators of long-term functional outcomes of early childhood brain injury. JAMA Pediatr. 170, 343–349. 10.1001/jamapediatrics.2015.4485 26902662 PMC5488264

[B56] Weaver S. M. Chau A. Portelli J. N. Grafman J. (2012). Genetic polymorphisms influence recovery from traumatic brain injury. Neuroscientist. 18, 631–644. 10.1177/1073858411435706 22402485

[B57] White T. E. Ford G. D. Surles-Zeigler M. C. Gates A. S. Laplaca M. C. Ford B. D. (2013). Gene expression patterns following unilateral traumatic brain injury reveals a local pro-inflammatory and remote anti-inflammatory response. BMC Genomics. 14:282. 10.1186/1471-2164-14-282 23617241 PMC3669032

[B58] Willmott C. Ponsford J. McAllister T. W. Burke R. (2013). Effect of COMT Val158Met genotype on attention and response to methylphenidate following traumatic brain injury. Brain Inj. 27, 1281–1286. 10.3109/02699052.2013.809553 23924290

[B59] Witcher K. G. Eiferman D. S. Godbout J. P. (2015). Priming the inflammatory pump of the CNS after traumatic brain injury. Trends Neurosci. 38, 609–620. 10.1016/j.tins.2015.08.002 26442695 PMC4617563

[B60] Yeates K. O. Taylor H. G. Walz N. C. Stancin T. Wade S. L.Yeates, K. O. Taylor H. G. Walz N. C. Stancin T. Wade S. L. (2010). The family environment as a moderator of psychosocial outcomes following traumatic brain injury in young children. Neuropsychology. 24, 345–356. 10.1037/a0018387 20438212 PMC2976589

